# Application of laboratory and portable attenuated total reflectance infrared spectroscopic approaches for rapid quantification of alpaca serum immunoglobulin G

**DOI:** 10.1371/journal.pone.0179644

**Published:** 2017-06-26

**Authors:** Ibrahim Elsohaby, Jennifer B. Burns, Christopher B. Riley, R. Anthony Shaw, J. Trenton McClure

**Affiliations:** 1 Department of Health Management, Atlantic Veterinary College, University of Prince Edward Island, Charlottetown, Prince Edward Island, Canada; 2 Department of Animal Medicine, Division of Infectious Diseases, Faculty of Veterinary Medicine, Zagazig University, Zagazig City, Sharkia Province, Egypt; 3 Institute of Veterinary, Animal and Biomedical Sciences, Massey University, Palmerston North, New Zealand; 4 National Research Council of Canada, Medical Devices Portfolio, Winnipeg, Manitoba, Canada; Kermanshah University of Medical Sciences, ISLAMIC REPUBLIC OF IRAN

## Abstract

The objective of this study was to develop and compare the performance of laboratory grade and portable attenuated total reflectance infrared (ATR-IR) spectroscopic approaches in combination with partial least squares regression (PLSR) for the rapid quantification of alpaca serum IgG concentration, and the identification of low IgG (<1000 mg/dL), which is consistent with the diagnosis of failure of transfer of passive immunity (FTPI) in neonates. Serum samples (n = 175) collected from privately owned, healthy alpacas were tested by the reference method of radial immunodiffusion (RID) assay, and laboratory grade and portable ATR-IR spectrometers. Various pre-processing strategies were applied to the ATR-IR spectra that were linked to corresponding RID-IgG concentrations, and then randomly split into two sets: calibration (training) and test sets. PLSR was applied to the calibration set and calibration models were developed, and the test set was used to assess the accuracy of the analytical method. For the test set, the Pearson correlation coefficients between the IgG measured by RID and predicted by both laboratory grade and portable ATR-IR spectrometers was 0.91. The average differences between reference serum IgG concentrations and the two IR-based methods were 120.5 mg/dL and 71 mg/dL for the laboratory and portable ATR-IR-based assays, respectively. Adopting an IgG concentration <1000 mg/dL as the cut-point for FTPI cases, the sensitivity, specificity, and accuracy for identifying serum samples below this cut point by laboratory ATR-IR assay were 86, 100 and 98%, respectively (within the entire data set). Corresponding values for the portable ATR-IR assay were 95, 99 and 99%, respectively. These results suggest that the two different ATR-IR assays performed similarly for rapid qualitative evaluation of alpaca serum IgG and for diagnosis of IgG <1000 mg/dL, the portable ATR-IR spectrometer performed slightly better, and provides more flexibility for potential application in the field.

## Introduction

Immunoglobulins are glycoproteins produced by B-lymphocytes, and are a crucial component of the host’s adaptive immune system [1]. Camelids have an epitheliochorial microcotyledonary placenta that does not allow the transplacental transfer of immunoglobulins from the dam to the fetus [2]. Therefore, newborn camelids are born essentially hypogammaglobulinemic and rely on the transfer of immunoglobulins through colostrum intake and enteric absorption for passive immunity [2, 3]. Immunoglobulin G (IgG) is the predominant class of colostral immunoglobulins involved in the transfer of passive immunity to newborn crias [4]. Inadequate transfer of IgG (<1000 mg/dL) to neonatal crias is know as failure of transfer of passive immunity (FTPI) and is associated with increased incidences of infections including septicemia, diarrhea, pneumonia, arthritis, omphalitis and meningitis [5–7]. The reported prevalence of FTPI in neonatal camelids in the United States is 21% [8]. Therefore, early and accurate diagnosis of FTPI in camelids is an integral part of most camelid husbandry programs that can reduce morbidity and mortality rates for crias [9].

Several diagnostic tests are available for measuring serum IgG concentration and assessing FTPI in llamas and alpacas including: radial immunodiffusion (RID) assay, immunoturbidimetric assay, gamma-glutamyl transferase, zinc sulfate turbidity, glutaraldehyde coagulation, sodium sulfate precipitation, and refractometry [8, 10, 11]. Although RID is the historical direct and quantitative reference method for measuring IgG concentrations, it has significant drawbacks including high cost, time required to obtain results (18–24 h), requirements for a high level of technical skills to perform, utilization of labile reagents, and poor adaptation to field settings [12, 13]. The other methods described above have been used for measuring camelid IgG concentrations and the diagnosis of FTPI with varying degrees of accuracy [8, 14]. However, data supporting the use of these assays are sparse. Thus, there is still a requirement for rapid, accurate, and automated methods to quantify camelid serum IgG concentration and diagnose FTPI.

Infrared (IR) spectroscopy is recognized as an analytical tool suitable for qualitative and quantitative determination of individual components within complex biological samples including serum, plasma, milk and urine [15–17]. IR spectroscopy is simple, rapid, accurate and requires minimal or no sample preparation [18, 19]. Modern computing technology has catalyzed the adoption of IR spectroscopy as a routine practical analytical/diagnostic tool by integration with chemometric tools such as partial least squares regression (PLSR) and principal component analysis (PCA) [20]. The most common IR spectroscopic sampling techniques are transmission and attenuated total reflectance infrared (ATR-IR) methods [21, 22]. Technical difficulties commonly encountered with transmission techniques include practical issues associated with filling and cleaning short-path length cells (for liquid samples), uncertainties in optical path length (for dried films), and the time required for sample submission and preparation [21, 22]. In comparison, ATR-IR spectroscopy by its nature does not have issues associated with optical path length or sample thickness. Previous reports have illustrated the use of transmission-IR spectroscopy in combination with PLSR to quantify IgG concentrations in bovine [23, 24], equine [25] and camelid species [26]. However, it is less amenable for field use on the farm, veterinary clinics or small laboratories. Miniaturization of the IR spectroscopy components has allowed the development of small compact laboratory and portable IR spectrometers that can be used in field settings, while offering simplicity, selectivity, and performance similar to that of the benchtop instruments [27]. The objective of the present study was to investigate the feasibility and compare the performance of laboratory grade and portable ATR-IR spectrometers combined with multivariate analysis (PLSR) for the rapid quantification of alpaca serum IgG concentrations and the identification of low IgG (<1000 mg/dL), which is consistent with a diagnosis of FTPI in neonates.

## Materials and methods

### Serum samples

Serum samples from 175 privately owned alpacas were used. These were collected between 2009 and 2011 from six farms in Ontario and New Brunswick, Canada (n = 82) and three farms in South Australia, Australia (n = 93). Age and sex data were only available for 129/175 alpacas. Of these 129 alpacas, 81 were female and 48 were male. Of the 129 animals with data on their ages, four alpacas were <2 months of age, 5 were 2–3 months of age, 15 were 3–6 months of age, 12 were between 6 months and 1 year of age, and 93 were >1 year of age. Blood samples were collected from alpacas via jugular venipuncture into a sterile, plastic vacutainer tube without anticoagulant, then centrifuged at 1500 g for 10 min at room temperature. These serum samples were collected for a previous study [26] and were stored frozen at −80°C at the University of Prince Edward Island (UPEI). The research protocol was reviewed and approved by the UPEI Animal Care Committee and the Animal Ethics Committee of the University of Adelaide.

### Reference RID assay

Serum samples were left to thaw for 60 min at room temperature (20–24°C) and then vortexed at maximum of 2700 rpm for 10 s. The IgG concentrations of alpaca sera were measured by the reference method using a commercial camelid IgG RID kit (Triple J Farms; Bellingham, WA, USA) [26]. Serum samples and RID standards were tested undiluted in replicates of five. The average of the assay standards was used to build a RID calibration curve that was then used to determine the IgG concentration of each sample. Serum samples with an IgG concentration greater than the manufacturer’s upper standard value (>3000 mg/dL) were diluted (1:1 or 1:2) with 0.85% saline and retested.

### ATR-IR spectroscopy

**Fig 1:**
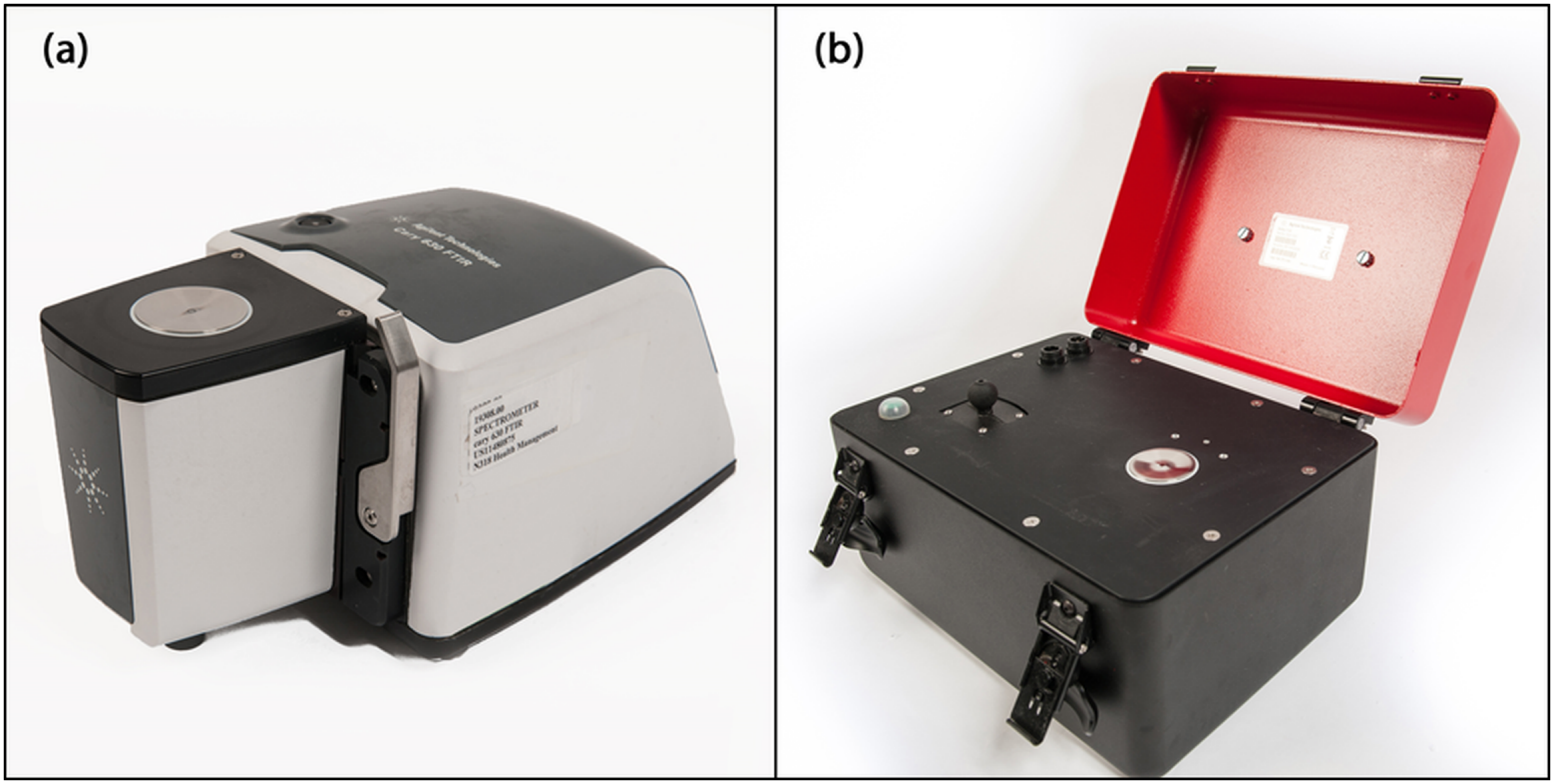
Attenuated total reflectance infrared spectrometers. (a) laboratory Cary 630 and (b) portable 4500 series (Agilent Technologies, Dansbury, Connecticut, USA).

Infrared spectra were acquired using both laboratory Cary 630 (Fig 1a) and portable 4500 series (Fig 1b) attenuated total reflectance mid-infrared spectrometers (Agilent Technologies, Dansbury, Connecticut, USA). The laboratory Cary 630 spectrometer is interfaced with a customized triple-reflection diamond ATR sampling accessory with a 1-mm diameter sampling surface, and a 200 μm active area providing a 2 μm depth of penetration for IR energy at 1700 cm^-1^. However, the portable 4500 series spectrometer is interfaced with a triple-reflection diamond crystal ATR accessory with a 2-mm diameter sampling surface and a 200 μm active area providing a 6 μm effective penetration depth for IR energy at 1700 cm^-1^.

Thawed serum samples were diluted (1:1) with deionized sterile water and vortexed at a maximum of 2700 rpm for 10 s. From each diluted sample a 5 μL aliquot was placed onto the diamond crystal of the spectrometer and dried by a stream of air from a domestic hair dryer. Samples were fully dried within 3–5 min, forming a thin film on the crystal. The IR spectra were then collected using Agilent MicroLab PC software (Agilent Technologies, Dansbury, Connecticut, USA). At a spectral resolution of 8 cm^-1^, 512 scans were co-added to optimize the signal to noise ratio over the wavenumber range of 4000–650 cm^-1^. Two independent spectra were collected from each serum sample with each spectrometer. The spectrometer crystal was cleaned with 100% ethanol and a laboratory wipe between each sample application and a new background spectrum (I_0_) was performed before applying the next serum sample. A total of 350 (175 x 2) ATR-IR spectra were collected from each spectrometer, saved in spectrum (SPC) format, and then converted into printable (PRN) formatted data using GRAMS software (GRAMS/AI version 7.02, Thermo Fisher Scientific). The PRN format spectral data were imported into MATLAB^®^ (version R2016a, MathWorks, Natick, MA) for data analyses.

### Spectral pre-processing

Spectra from each spectrometer were subjected to several trial pre-processing methods prior to provisional PLSR analyses, including Savitsky-Golay smoothing (2^nd^ order polynomial function with 9 points), first-order and second-order derivatization [28] and two different normalization methods (Standard normal variate (SNV) and vector normalization) [29, 30], then followed by spectral region selection. Only the combined spectral regions 3700–2600 cm^-1^ and 1800–1300 cm^-1^ were selected for further analysis [26]. Subsequently, spectrum outlier detection was performed using Dixons Q-test [31, 32] at each wavenumber. Spectra were designated as outliers and excluded from further analysis if more than 50% of absorbance values were outside the 95% confidence level. The average of the duplicate spectra for each serum sample (after removal of outliers if applicable) was used for subsequent analysis.

### Calibration models development

A multivariate regression method (PLSR) was used to create calibration models for each ATR-IR spectrometer separately. The 175 serum samples were sorted based on their IgG concentration (lowest to highest) obtained from the reference RID based method and linked to their corresponding pre-processed spectra, and then split into test and calibration sets. The spectrum of every third serum sample was selected as a member of the test set (n = 58); the remaining samples (n = 117) were assigned to the calibration set. This ensured that the test set encompassed the full range of IgG values for evaluating the predictive performance of the calibration models. The calibration set (n = 117) was further randomly split into training (n = 59) and validation (n = 58) data sets for model development.

A previously described PLSR approach was applied to the training set to develop 30 trial calibration models with the number of PLS factors ranging from 1 to 30 [26]. Each trial model was used to quantify the IgG concentrations of the validation set of serum samples. This procedure was repeated 10,000 times, utilizing randomly assigned splits of the calibration data set into new training and validation sets. The root mean squared error for the Monte Carlo cross-validation value (RMMCCV) [33, 34] was calculated for each of the 30 trial calibration models, and the optimal number of PLS factors was chosen based on the lowest RMMCCV value. Once the number of PLS factors had been determined, the training and validation sets were recombined to build the final calibration model with the optimal number of PLS factors.

### Evaluation of trial calibration models

The predictive performance of each provisional calibration model was evaluated using the test data set. The level of agreement between IgG concentrations measured by the reference RID method and those predicted by the calibration models was assessed for both the calibration and test data sets by scatter plot, and determination of Pearson correlation coefficients [35]. The differences and interchangeability between the IgG measured by RID and ATR-IR methods for the test set were further evaluated by the Bland-Altman plot [36, 37].

The potential utility of each ATR-IR calibration model was further evaluated by calculation of the ratio of predictive deviation (RPD) and the range error ratio (RER) [38]. For RPD values <2, the calibration model was considered to be poorly predictive, those between 2.0 and 2.5 as adequate for qualitative evaluation or for screening purposes, values >2.5 (or RER >10) were regarded as acceptable for quantification, and values >3 (or RER >20) were considered that the calibration model was suitable for accurate quantitative analysis [38].

The practical applicability of the ATR-IR calibration models for the diagnosis of FTPI (serum IgG <1000 mg/dL) in alpaca serum was evaluated. The diagnostic sensitivity, specificity and accuracy were calculated using 2 x 2 tables (diagt command in Stata vs 14.0 statistical software, StataCorp, College Station, TX) for both the test set and entire data set. Sensitivity (Se) was defined as the proportion of samples with IgG <1000 mg/dL, as determined by RID, that was correctly classified as positive by the ATR-IR assay. Conversely, specificity (Sp) was defined as the proportion of samples with IgG >1000 mg/dL that was correctly classified as negative by the ATR-IR assay. Accuracy was defined as the percentage of all samples that were correctly classified by the ATR-IR assay.

## Results

### Serum IgG determined by RID method

The RID-determined IgG concentrations of the 175 alpaca serum samples ranged from 394 to 6327 mg/dL, with an average of 2654 mg/dL and standard deviation of 1305 mg/dL. The mean, SD and range of the RID-determined IgG concentration for the calibration and test data sets are presented in Table 1.

**Table 1:** Descriptive statistics for the 175 alpaca serum samples in the calibration and test data sets (RID-determined serum IgG values).

Datasets	RID-IgG concentration (mg/dL)			
	n	Mean	SD	Range
Calibration set	117	2665	1320	5934
Test set	58	2632	1285	5172

### ATR-IR spectra

**Fig 2:**
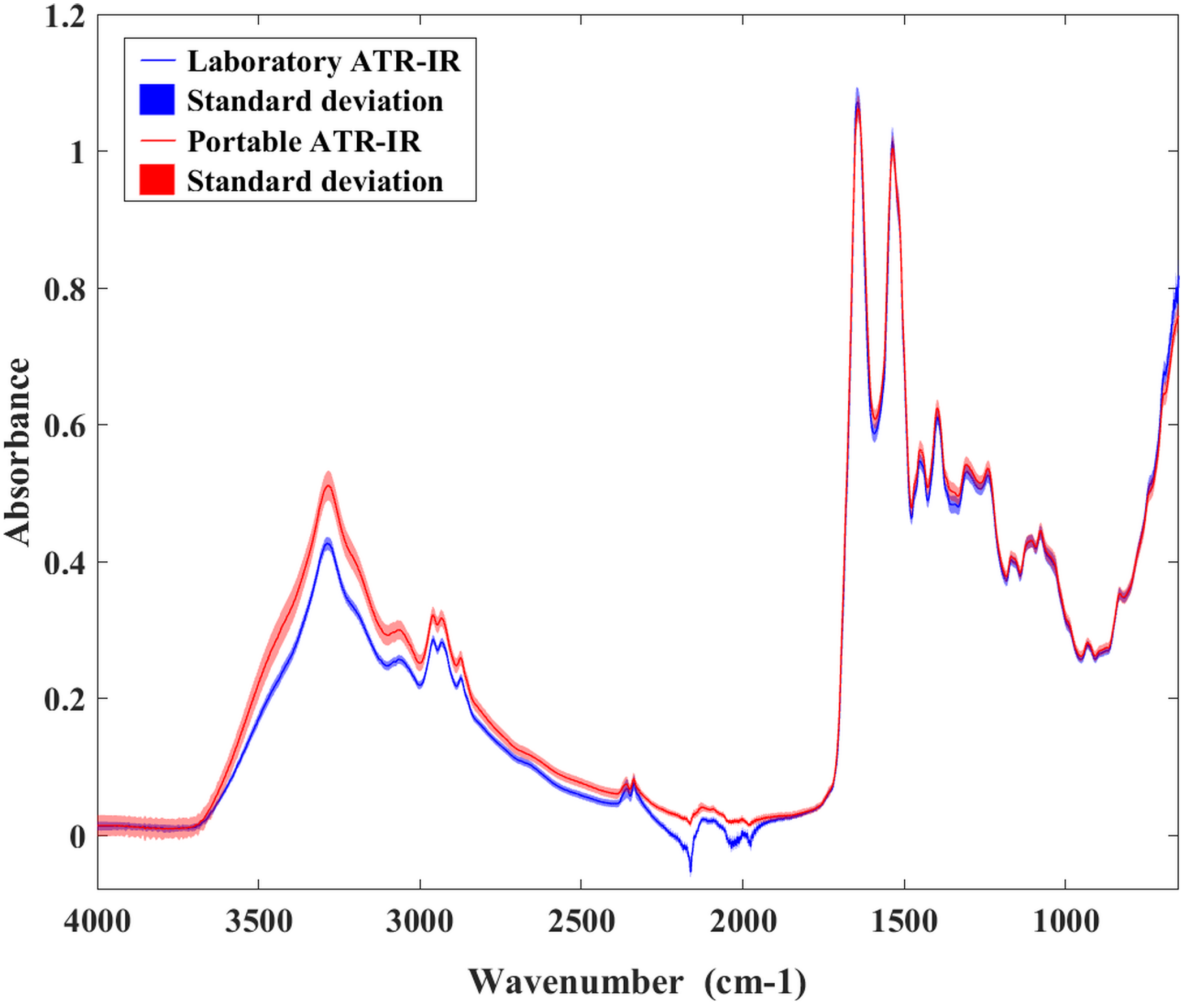
The average reflectance spectra and their corresponding standard deviation values of alpaca serum samples obtained from the laboratory and portable ATR-IR spectrometers.

The IR spectrum of alpaca serum collected from both the laboratory (Cary 630) and portable (4500 series) ATR-IR spectrometers over the wavenumber range of 4000–650 cm^−1^ showed distinct bands characteristic of functional group vibrations (Fig 2). A broad strong absorption band centered at 3300 cm^−1^ was associated with N–H stretching vibration (Amide A) of the protein amide linkages, and strong absorptions centered around 1650 and 1550 cm^−1^ were associated with protein C = O stretching (Amide I) and N–H bending (Amide II) vibrations, respectively [15, 18]. Both laboratory and portable ATR-IR spectrometers provided similar spectral patterns but the portable ATR-IR generated slightly higher signal intensities compared to the laboratory grade spectrometer, due to a slightly longer effective optical pathlength for that particular ATR element/geometry (Fig 2). The 2200–1900 cm^−1^ range is compromised in both systems due to very strong absorptions by the diamond coating on the ATR elements. However, the effect was most clearly evident for the Cary 630 spectra.

### Calibration models

**Fig 3:**
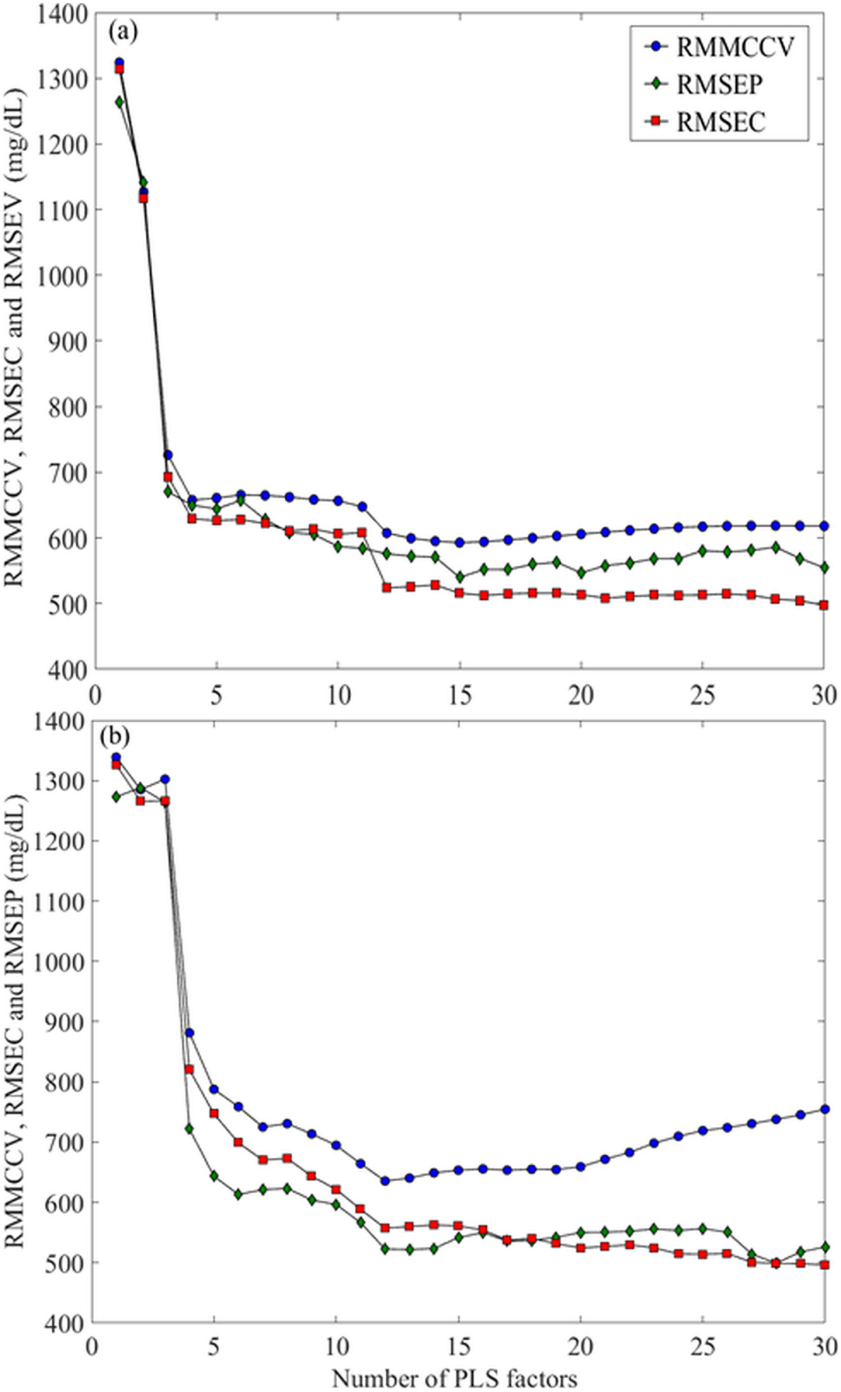
Plots of RMMCCV, RMSEC and RMSEP against the number of partial least squares (PLS) factors. The optimum number of PLS factors for (a) laboratory grade and (b) portable attenuated total reflectance infrared (ATR-IR) assays were determined to be 15 and 12, respectively, based on the lowest RMMCCV.

The parameters and performance characteristics of the PLS calibration models that were built using a different combination of spectral pre-processing techniques are presented in Table 2. The optimal PLS calibration models for the laboratory grade and portable ATR-IR spectrometers were obtained using data from first order derivatives spectra and vector normalization, and smoothed spectra and vector normalization, respectively. The optimum number of PLS factors for these models were 15 for the laboratory grade ATR-IR (Fig 3a) and 12 for the portable ATR-IR spectra (Fig 3b), based on the lowest IgG RMMCCV (592 and 635 mg/dL, respectively). Fig 3 displays the root mean squared error of calibration (RMSEC) and root mean squared error of prediction (RMSEP) plotted against the number of PLS factors.

**Table 2:** Comparison of analytical methods based upon various spectral pre-processing protocols.

Pre-processing	PLS factors	Calibration (n = 117)			Prediction (n = 58)			
		RMMCCV	*r*	RMSEC	*r*	RMSEP	RPD	RER
		(mg/dL)		(mg/dL)		(mg/dL)		
**Laboratory ATR-IR**								
Smoothing (9 points)	6	669	0.95	630	0.90	639	2	8.1
Smoothing + normalization (SNV)	6	671	0.94	540	0.90	578	2.2	8.9
Smoothing + vector normalization	6	678	0.95	638	0.91	647	2	8
1^st^ derivatives (9 points)	15	592	0.95	515	0.91	545	2.4	9.5
1^st^ derivatives + normalization (SNV)	15	595	0.95	518	0.90	541	2.4	9.6
1^st^ derivatives + vector normalization	15	592	0.95	516	0.91	540	2.4	9.6
2^nd^ derivatives (9 points)	18	618	0.94	531	0.88	618	2.1	8.4
2^nd^ derivatives + normalization (SNV)	18	630	0.94	533	0.88	615	2.1	8.4
2^nd^ derivatives + Vector normalization	18	635	0.94	527	0.87	608	2.1	8.5
**Portable ATR-IR**								
Smoothing (9 points)	12	628	0.95	551	0.91	527	2.4	9.8
Smoothing + normalization (SNV)	12	649	0.94	564	0.92	548	2.3	9.4
Smoothing + vector normalization	12	635	0.95	557	0.91	523	2.5	9.9
1^st^ derivatives (9 points)	16	613	0.95	509	0.92	563	2.3	9.2
1^st^ derivatives + normalization (SNV)	16	611	0.95	508	0.92	552	2.3	9.4
1^st^ derivatives + vector normalization	16	610	0.95	506	0.92	551	2.3	9.4
2^nd^ derivatives (9 points)	17	656	0.95	520	0.93	576	2.2	9
2^nd^ derivatives + normalization (SNV)	17	684	0.95	526	0.92	583	2.2	8.9
2^nd^ derivatives + Vector normalization	17	668	0.95	529	0.92	578	2.2	9

PLS = Partial least squares; RMMCCV = Root mean squared error of the Monte Carlo cross validation value; *r* = Pearson correlation coefficient; RMSEC = Root mean squared error of calibration; RMSEP Root mean squared error of prediction; RPD (ratio of predictive deviation) = SD divided by RMSEP; RER (range error ratio) = Range divided by RMSEP; SNV Standard normal variate.

### Calibration models performance

**Fig 4:**
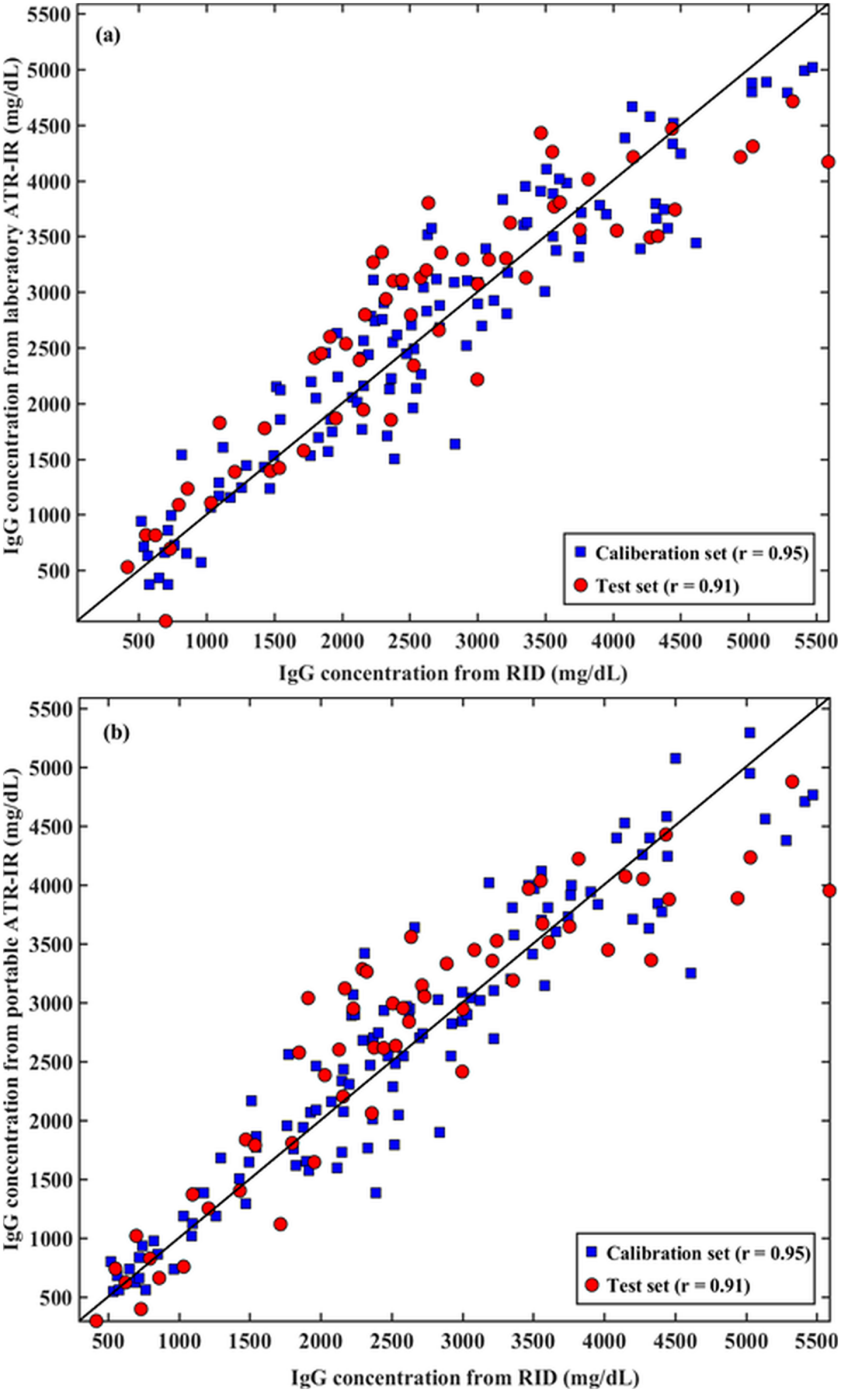
Scatter plots comparing Immunoglobulin G (IgG) measured by the reference radial immunodiffusion (RID) assay to those provided by (a) laboratory grade and (b) portable ATR-IR based assays for the calibration and test sets.

**Fig 5:**
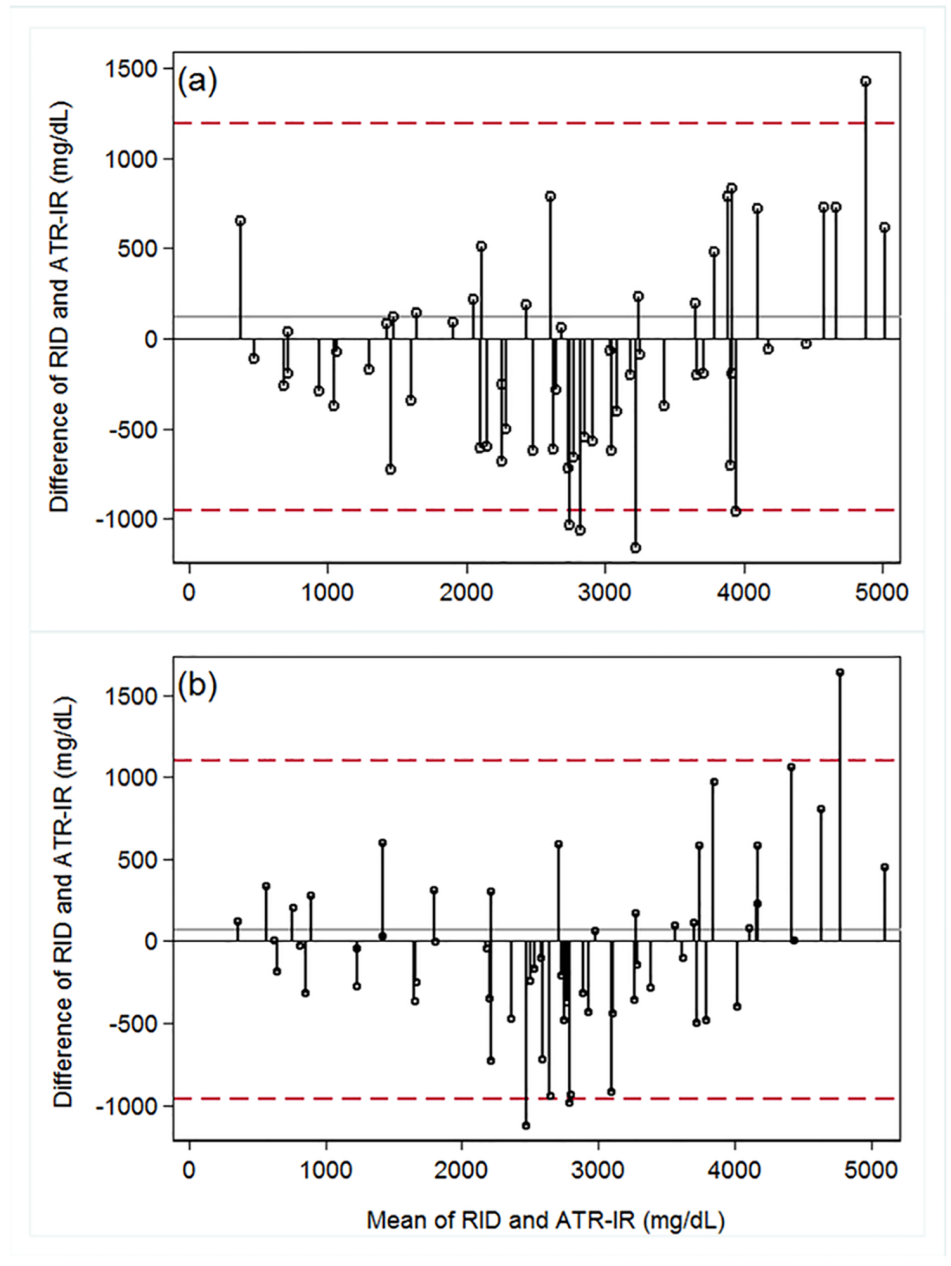
Bland-Altman plots for the test (n = 58) data set. (a) for laboratory ATR-IR assay, the dashed lines represent the 95% confidence limits of agreement (– 950 to 1191 mg/dL), while the solid line represents the mean difference between laboratory ATR-IR and RID assays (120.5 mg/dL). (b) for portable ATR-IR assay, the dash lines represent the 95% confidence limits of agreement (– 960 to 1101 mg/dL), while the solid line represents the mean difference between portable ATR-IR and RID assays (71 mg/dL).

Scatter plots (Fig 4) show the level of agreement between serum IgG concentrations measured by the reference RID assay and those predicted by both the laboratory grade (Fig 4a) and portable (Fig 4b) ATR-IR based assays. For both laboratory grade and portable ATR-IR spectrometers, the Pearson correlation coefficient (*r*) for the calibration data set was 0.95; corresponding value for the test data set was 0.91. The Bland-Altman plots (Fig 5) revealed that the average of the differences between RID-determined IgG concentration and the laboratory ATR-IR assay was 121 mg/dL (Fig 5a), and 71 mg/dL for the portable ATR-IR assay (Fig 5b), indicating no signficant bias between these methods. The 95% confidence interval for laboratory grade and portable ATR-IR spectrometers ranged from –950 to 1191 mg/dL and –960 to 1101 mg/dL, respectively. The RPD and RER values for laboratory grade and portable ATR-IR based assays were 2.4 and 9.6, and 2.5 and 9.9, respectively, indicating that both ATR-IR assays were acceptable for qualitative evaluation or for screening of alpaca serum IgG concentrations.

The diagnostic test characteristics (Se, Sp and accuracy) of the laboratory and portable ATR-IR assays for diagnosis of serum IgG <1000 mg/dL (FPTI cut-off) were determined separately for the test sets and for the entire data sets (Table 3). Within the whole data set, 21 out of 175 serum samples had RID-IgG concentration <1000 mg/dL, resulting in a true low IgG prevalence of 12%. The number of serum samples that had IgG concentrations <1000 mg/dL according to the laboratory grade (n = 18) and portable (n = 21) ATR-IR based assays, resulted in apparent prevalences of 10% and 12%, respectively. For the laboratory grade ATR-IR based assay, there were no false positives and three false negatives identified. For the portable ATR-IR based assay, one false positive and one false negative were identified (Table 3).

**Table 3:** Diagnostic test characteristics for assessment of IgG <1000 mg/dL, the cut-off for failure of transfer of passive immunity (FTPI).

Dataset	n	Test characteristics						
		TP	FP	TN	FN	Se	Sp	Accuracy
**Laboratory ATR-IR**								
Test set	58	5	0	51	2	71%	100%	97%
All data	175	18	0	154	3	86%	100%	98%
**Portable ATR-IR**								
Test set	58	6	1	50	1	86%	98%	97%
All data	175	20	1	153	1	95%	99%	99%

n = number of samples; TP = true positives; FP = false positives; TN = true negatives; FN = false negatives; Se = sensitivity; Sp = specificity.

## Discussion

This study showed that IR spectroscopy, using either a laboratory grade or portable ATR-IR spectrometer, is an adequate method for rapid qualitative evaluation and for screening of alpaca serum IgG concentrations. The performance of the IR-based analytical methods depended marginally on the choice of IR spectrometer, and more critically on spectral pre-processing strategies. In the present study, the IR-based assays showed performance comparable to those previously reported for transmission-IR spectroscopy [26]. In contrast to transmission spectroscopy, the ATR-IR based approaches have the advantage of reduced sample preparation time (<5 min), no requirement for a transmission substrate, and reduced imprecision associated with variability in sample volume and film thickness, issues that can contribute to imprecision in transmission-IR based assays [24, 39].

The optimal analytical methods were confirmed as adequate for qualitative evaluation or for screening of alpaca IgG concentration by both their predictive accuracy (i.e., RMSEP) and by standard tests to characterise PLSR-based calibrations (RPD and RER values) [38]. Spectral derivation and smoothing contributed to optimization of the analytical methods using the laboratory grade and portable spectrometers, respectively (Table 2). Similar results were previously reported in related studies that used either transmission or ATR-IR spectrometers [24, 26]. Whether smoothed or derivatized spectra were used, vector normalization proved more beneficial for prediction of IgG concentration in alpaca serum than standard normal variate (SNV). Interestingly, the opposite behaviour was observed previously for analogous transmission IR-based IgG assays [26]. The optimum PLS model for the portable ATR-IR based assay required fewer PLS-factors than did that of the laboratory ATR-IR based assay (Fig 3). This could be attributed to better coupling of the optical element within the optical train for the dedicated ATR (portable 4500 series) instrument as compared to the ATR module within the laboratory spectrometer [40]. Further, the larger number of PLS-factors suggests that the laboratory ATR-IR assay may be less robust than the portable ATR-IR counterpart, by virtue of its reliance upon additional factors that are relatively noisy [41].

The portable ATR-IR assay showed higher utility (higher RPD and RER values), and more flexibility for field application [40, 41] than the laboratory grade ATR-IR assays reported in the present study, and that previously reported for bovine serum IgG [24]. Correlation between the RID-based IgG analyses and ATR-IR based assays was better than that previously reported for transmission-IR based IgG assays for alpaca [26] and equine [25] sera, but worse than that achieved for transmission and ATR-IR based bovine IgG assays [24]. Limitations in the predictive ability of both laboratory grade and portable ATR-IR assays in this study may be attributed to the source of the samples. Serum samples were collected from two different geographical regions and the IgG content was highly variable, ranging from 394 to 6327 mg/dL. Further, only 12% (21 out 175) of serum samples having IgG concentration less than 1000 mg/dL. As a result, the developed assays were weighted toward higher IgG concentrations.

The correlation with RID-IgG concentrations was lower than that reported for comparison of RID-IgG to immunoturbidimetric methods [10], but higher than that reported for comparisons of serum total protein refractometry versus RID-IgG [8]. The Bland-Altman plots (Fig 5) revealed a non-significant bias for the portable ATR-IR-based assay as compared to the lab system counterpart. However, the bias for both ATR-IR based assays was higher than that previously reported (20.2 mg/dL) for the transmission-IR based assay [26].

Both ATR-IR assays showed excellent Sp, but the Se of the portable ATR-IR assay was higher than that of the laboratory grade ATR-IR assay (Table 3). When compared with reported Se and Sp of other methods available to assess FTPI in neonatal camelids, these results are equivalent to or better than most [8, 10, 26, 42]. Both ATR-IR spectroscopic assays correctly classified the majority of FTPI cases as such, and the misclassified samples had RID IgG concentrations very close to the diagnostic cut-off of 1000 mg/dL. This suggests that the likelihood of misdiagnosis is small; the false negative samples correspond to only partial FTPI, with minimal risk of morbidity and mortality [2, 7].

Indeed, the transferability into field conditions (veterinary clinics and large farms) remains open for discussion and a further in depth investigation is needed to better evaluate the portable ATR-IR assay for rapid field evaluation of FTPI. However, this study is a proof of concept, with respect to the emerging technologies for automated measurement of alpaca serum IgG concentration and diagnosis of FTPI.

## Conclusions

This study supports the applicability of both laboratory grade and portable ATR-IR spectroscopy as the basis for rapid qualitative evaluation of alpaca serum IgG concentration and diagnosis of FTPI. While the laboratory grade and portable ATR-IR based assays perform similarly, the portable ATR-IR assay has the potential advantage of enabling determination of IgG concentration in the field.
